# Emerging highly pathogenic avian influenza (H5N8) virus in migratory birds in Central China, 2020

**DOI:** 10.1080/22221751.2021.1956372

**Published:** 2021-07-30

**Authors:** Jiasong Xiong, Hao Zhou, Lifang Fan, Gongliang Zhu, Yong Li, Guang Chen, Jun Zhang, Jundong Li, Hesong Zheng, Wei Feng, Jing Chen, Guoxiang Yang, Quanjiao Chen

**Affiliations:** a CAS Key Laboratory of Special Pathogens and Biosafety, Wuhan Institute of Virology, CAS Center for Influenza Research and Early Warning, Center for Biosafety Mega-Science, Chinese Academy of Sciences, Wuhan, People’s Republic of China; b University of Chinese Academy of Sciences, Beijing, People’s Republic of China; c Department of pathology, Hubei Cancer Hospital, Wuhan, People’s Republic of China; d The Monitoring Center of Wildlife Diseases and Resource of Hubei Province, Wuhan, People’s Republic of China; e Longgan Lake National Nature Reserve Administration, Wuhan, People’s Republic of China; f Wang Lake Wetland Nature Reserve Administration of Huangshi City, Huangshi, People’s Republic of China

**Keywords:** HPAIV H5N8, phylogenetic analysis, migratory birds, histopathology, flyway

## Abstract

Eleven highly pathogenic avian influenza H5N8 viruses (clade 2.3.4.4b) were detected in migratory birds in Central China between November and December 2020, which were highly homologous to strains isolated in Europe from October to December 2020. Phylogenetic analysis indicated that the strains in the study possibly spread from Siberia by migratory birds. In this study, we found H5N8 virus infection in migratory birds could cause severe pathological damage and high viral load in multiple organs.

## Dear Editor

Since the H5N1 highly pathogenic avian influenza virus (HPAIV) was first isolated from geese in Guangdong province, China in 1996 [1], H5 HPAIVs have continued to evolve and have subsequently been divided into 10 clades (clades 0–9) [2]. HPAIV H5N8 was first detected in domestic ducks in eastern China in 2010 [3]. In 2014, clade 2.3.4.4 HPAIVs H5N8 caused outbreaks in Korea [4], and subsequently, clade 2.3.4.4a HPAIV H5N8 spread to North America and Europe via migratory birds [5,6]. During 2016–2017, clade 2.3.4.4b HPAIVs H5N8 were found in Asia, which then rapidly transmitted to Europe and Africa. The introduction of H5N8 in Europe during 2016–2017 led to the largest outbreak of H5 HPAIVs, affecting more than 40 countries, resulting in thousands of outbreaks in poultry and wild birds [7]. During 2017–2019, there were sporadic reports of clade 2.3.4.4b HPAIVs H5N8 in poultry and wild birds in Europe, Africa, and the Middle East [8]. From 16 August 2020 to 7 December 2020, a new massive HPAIVs H5N8 outbreak in Eurasia was reported including 21 Eurasian countries [9].

From 4 November 2020, samples of 21 migratory birds collected by the monitoring centre of wildlife diseases and resource of Hubei province were successively sent to our laboratory. Birds included 17 tundra swans (*Cygnus columbianus*) (13 dead), 3 bean geese (*Anser fabalis*) (2 dead), and 1 whiskered tern (*Chlidonias hybrida*) (dead), from the Longgan Lake National Nature Reserve, Wang Lake, and Xisai Mountain, respectively (Appendix Figure 1, Appendix Table 1). Samples included oropharyngeal swabs, cloacal swabs, lung, liver, heart, spleen, and kidney. Among the 21 birds, 11 were H5N8 positive (Appendix Table 1), from which 8 samples were sequenced using next-generation sequencing. The whole genome of the eight samples was obtained. Nucleotide homology analysis revealed that the whole genome of the eight strains shared high similarities with each other (99.1–100%). All of the segments of the eight strains were blasted in NCBI and GISAID, and the strains with the highest homology for the eight segments were found to be strains isolated from October to December 2020 in Europe (99.15–99.90%) (Appendix Table 2).

**Figure 1.**
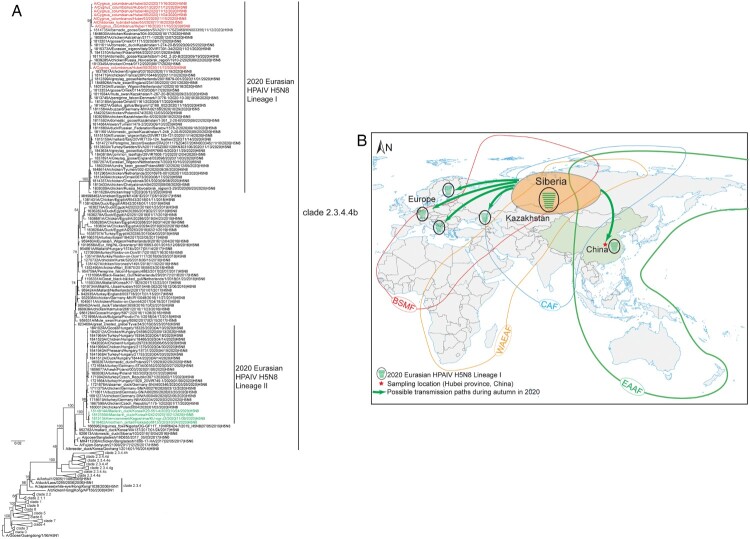
The maximum-likelihood phylogenetic analyses of HPAIVs H5N8 in migratory birds in Hubei province, Central China and the proposed transmission routes of the H5N8 viruses in this study. (A) Maximum-likelihood phylogenetic tree of the HA gene. H5N8 viruses in the study are indicated in red. H5N8 viruses collected in Korea and Japan during 2020–2021 are marked in green. The bootstraps of the main clade are signed. (B) The proposed transmission routes of the H5N8 viruses in this study. The HPAIVs H5N8 in this study spread from Siberia to Central China by migratory birds along East Asian-Australasian Flyway in autumn. Siberia is coloured orange in oval. EAAF, East Asian-Australasian Flyway; CAF, Central Asian Flyway; WAEAF, West Asian-East African Flyway; BSMF, Black Sea/Mediterranean Flyway; HPAIV, Highly pathogenic avian influenza virus; HA, haemagglutinin.

To further explore the source of these H5N8 viruses, maximum-likelihood and maximum clade credibility (MCC) phylogenetic trees were constructed. The maximum-likelihood phylogenetic tree of HA showed that all eight strains clustered with those isolated from Europe, Northern Kazakhstan, and Southwestern Russia (Siberia) collected from August to December 2020 and were grouped into clade 2.3.4.4b (Figure 1(A)). The MCC tree of HA has the similar topology as that of maximum-likelihood tree (Appendix Figure 2D). MCC trees demonstrated that each segment of H5N8 viruses in this study clustered with isolates collected in Eurasia from August to December 2020 and formed 2020 Eurasian HPAIV H5N8 Lineage I (Appendix Figure 2). It is worth noting that HPAIV H5N8 viruses detected in Korea and Japan from 2020 to 2021 clustered with isolates collected in Europe (Germany, Poland, Hungary, Czech Republic, and Slovakia) in early 2020 and formed 2020 Eurasian HPAIV H5N8 Lineage II (Appendix Figure 2). The nucleotide homology between strains detected in Japan and Korea and those in our study ranged from 92–97.8% (PB2, 94–94.5%; PB1, 94–94.3%; PA, 92–92.6%; HA, 95.7–96%; NP, 95.1–95.5%; NA, 95.2–95.4%; MP, 97.6–97.8%; and NS, 94.8–95.5%).

The time of most recent common ancestor (tMRCA) of each segment of the HPAIVs H5N8 in the study and isolates collected in Siberia (in the northern hemisphere) ranged from May 2020 to July 2020 (Appendix Figure 2). The time corresponds with summer in 2020 in the northern hemisphere. For the bird migration pattern, Ramachandra et al. [10] reported that the most common pattern is flying north in the spring to breed in the temperate zone or Arctic (e.g. Siberia) in summer and returning south to wintering grounds (warmer regions) in the fall. Furthermore, many bird populations migrate long distances along a flyway. Wild birds migrate to Siberia in spring, where multiple migratory flyways converge. After breeding in summer, the birds migrate from Siberia to southern hemisphere or tropical area in autumn. Thus, Siberia plays a role in disseminating influenza virus. In Siberia, HPAIVs H5N8 (carried by wild birds in multiple flyways) spread with the bird migration and caused a pandemic in Europe, Asia, and Africa during 2016–2018 [11]. According to the migration patterns of wild birds and phylogenetic analyses in the study, it can be speculated that the H5N8 viruses in the study may be spread by wild birds from somewhere to Siberia in summer. Then, these viruses spread from Siberia to Central China by migratory birds along East Asian-Australasian Flyway in autumn (Figure 1(B)).

Subsequently, the key amino acid substitutions that contributed to enhanced replication or virulence in mammals and avians and cross-species transmission were analysed. The typical polybasic amino acid cleavage site (PLREKRRKR/G) of the HA protein indicated that all eight strains were HPAIVs. The T160A mutation which has been associated with an affinity for α-2,6-linked sialic acid receptor and increased transmission in guinea pigs was found in the HA protein (Appendix Table 3). For PB2, PB1, and PA, several amino acid sites associated with increased polymerase activity and enhanced pathogenicity in mammals were detected in the eight strains. Moreover, a few molecular markers that caused increased virulence in poultry and mammals were detected in M1 and NS1 (Appendix Table 3). Further analysis showed the strains we detected had the same molecular maker (mentioned above) as those of the 2016–2017 Eurasian HPAIVs H5N8. To explore the pathogenicity of the H5N8 viruses in wild birds, haematoxylin and eosin staining and quantification of viral load were performed on the organs (lung, liver, heart, spleen, and kidney) of four dead migratory birds (three tundra swans and one whiskered tern collected in Longgan Lake National Nature Reserve on 16 November 2020) (Appendix Figure 3). Histopathological analysis of migratory birds in the study indicated inflammation and necrosis in multiple organs that are consistent with HPAIV H5N8 infection (Appendix Figure 3). And there is no significant difference in histopathology between tundra swan and whiskered tern. Specifically, histopathological analysis of lung presented destruction of the air capillaries, decreased number of air capillaries, haemorrhage and inflammatory cell infiltration in air capillaries (Appendix Figure 3A). In the liver, inflammatory cells near the central vein and oedema in the space of Disse were observed (Appendix Figure 3B). Myocarditis was found in the heart and was characterized by severe necrosis of the myocardial fibres with inflammatory cell infiltration (Appendix Figure 3C). In the spleen, lymphocytes depletion, necrosis of lymphocytes, and amyloidosis were observed. Also, necrosis of lymphocytes was found in lymphoid follicle (Appendix Figure 3D). In the kidney, necrosis of renal tubular epithelial cells and severe interstitial haemorrhage with inflammatory cell infiltration were observed (Appendix Figure 3E). It is worth mentioning that pathological changes in these organs are partly masked by postmortem autolytic changes, because these wild birds had been dead for too long when they were found. Next, the virus-specific antigen nucleoprotein (NP) was detected in lung (Appendix Figure 3G, 3H). A high viral load (copies of influenza A virus in 1ug total RNA) was detected in lung (10^5.80^–10^7.54^), liver (rang: 10^5.22^–10^6.54^), heart (rang: 10^2.92^–10^9.11^), spleen (range: 10^4.55^–10^6.23^), and kidney(10^5.03^–10^6.75^) collected from the four dead birds, respectively (Appendix Figure 3F). Collectively, HPAIV H5N8 infection in migratory birds tested in the study could cause severe lesions, high viral burden in multiple organs, and death of birds.

## Conclusion

HPAIVs H5N8 were detected in migratory birds since migratory birds passed through Hubei province at the end of 2020. Combining phylogenetic analyses of viruses with the migration pattern of wild birds, it can be speculated that H5N8 viruses in the study may be disseminated from Siberia by migratory birds to Central China. H5N8 virus was detected in multiple organs with high viral loads, and inflammation and necrosis were observed in these organs. Meanwhile, several amino acid mutations with enhanced virulence in mammals and birds have been identified. It is worth mentioning that the T160A mutation in the HA protein preferentially binds to the human receptor and increased transmission ability in mammals was observed. Taken together, we need to strengthen AIV surveillance in wild birds and poultry and take extra precautions to avoid potential threats to poultry and human health caused by HPAIV H5N8.

## Supplementary Material

Appendix_Figure_3.tif

Appendix_Figure_2H.tif

Appendix_Figure_2G.tif

Appendix_Figure_2F.tif

Appendix_Figure_2E.tif

Appendix_Figure_2D.tif

Appendix_Figure_2C.tif

Appendix_Figure_2B.tif

Appendix_Figure_2A.tif

Appendix_Figure_1.tif

Appendix_Table_3.docx

Appendix_Table_2.docx

Appendix_Table_1.docx

Revised_Appendix_Clean.docx
