# Supplementary figures and images for: Emerging highly pathogenic avian influenza (H5N8) virus in migratory birds in Central China, 2020
Source: Emerg Microbes Infect. 2021 Jul 30;10(1):1503–6. doi: 10.1080/22221751.2021.1956372 (PMC8330791; doi:10.1080/22221751.2021.1956372)

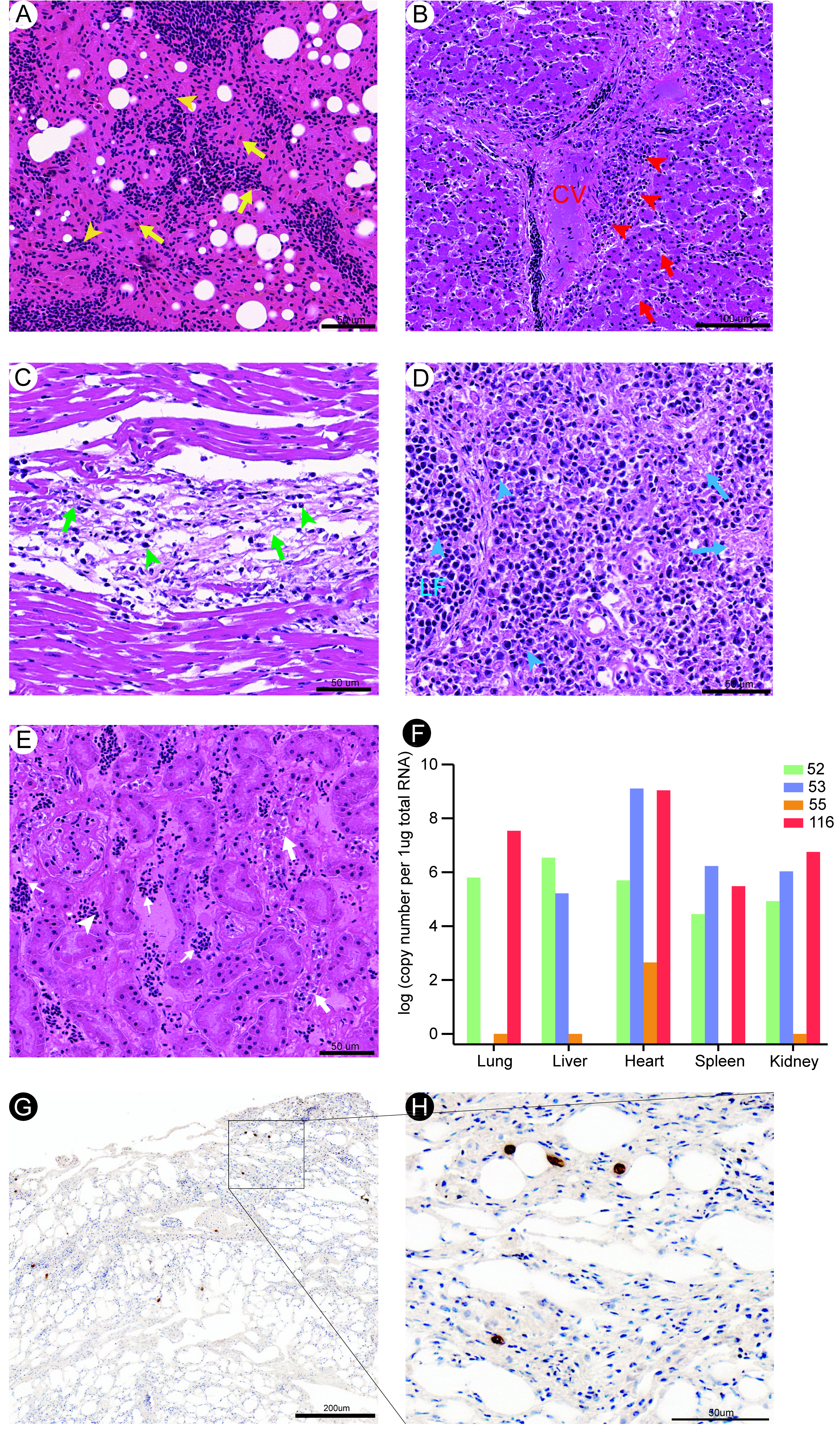

Supplement: Appendix_Figure_3.tif [file TEMI_A_1956372_SM8253.tif]

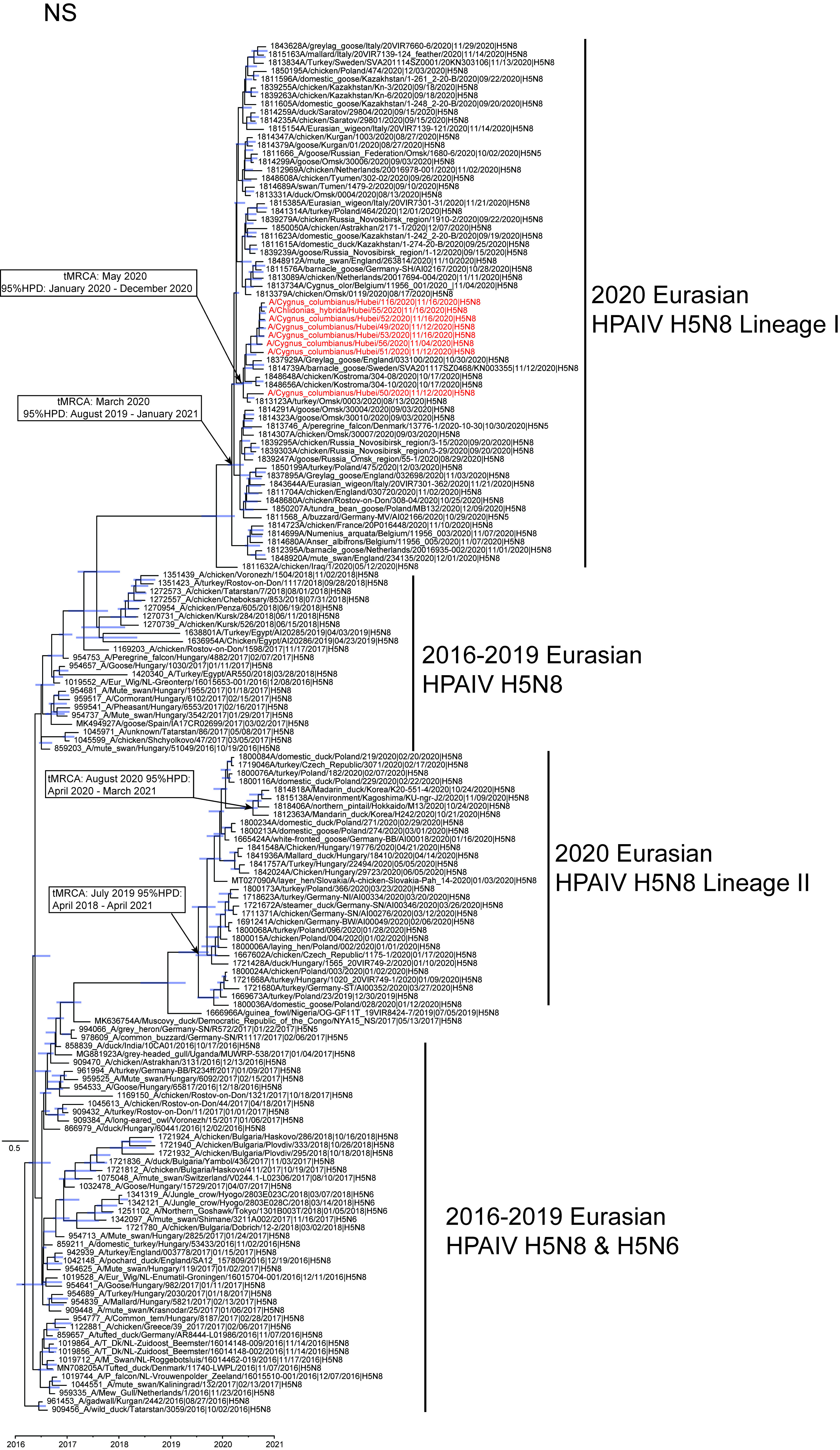

Supplement: Appendix_Figure_2H.tif [file TEMI_A_1956372_SM8252.tif]

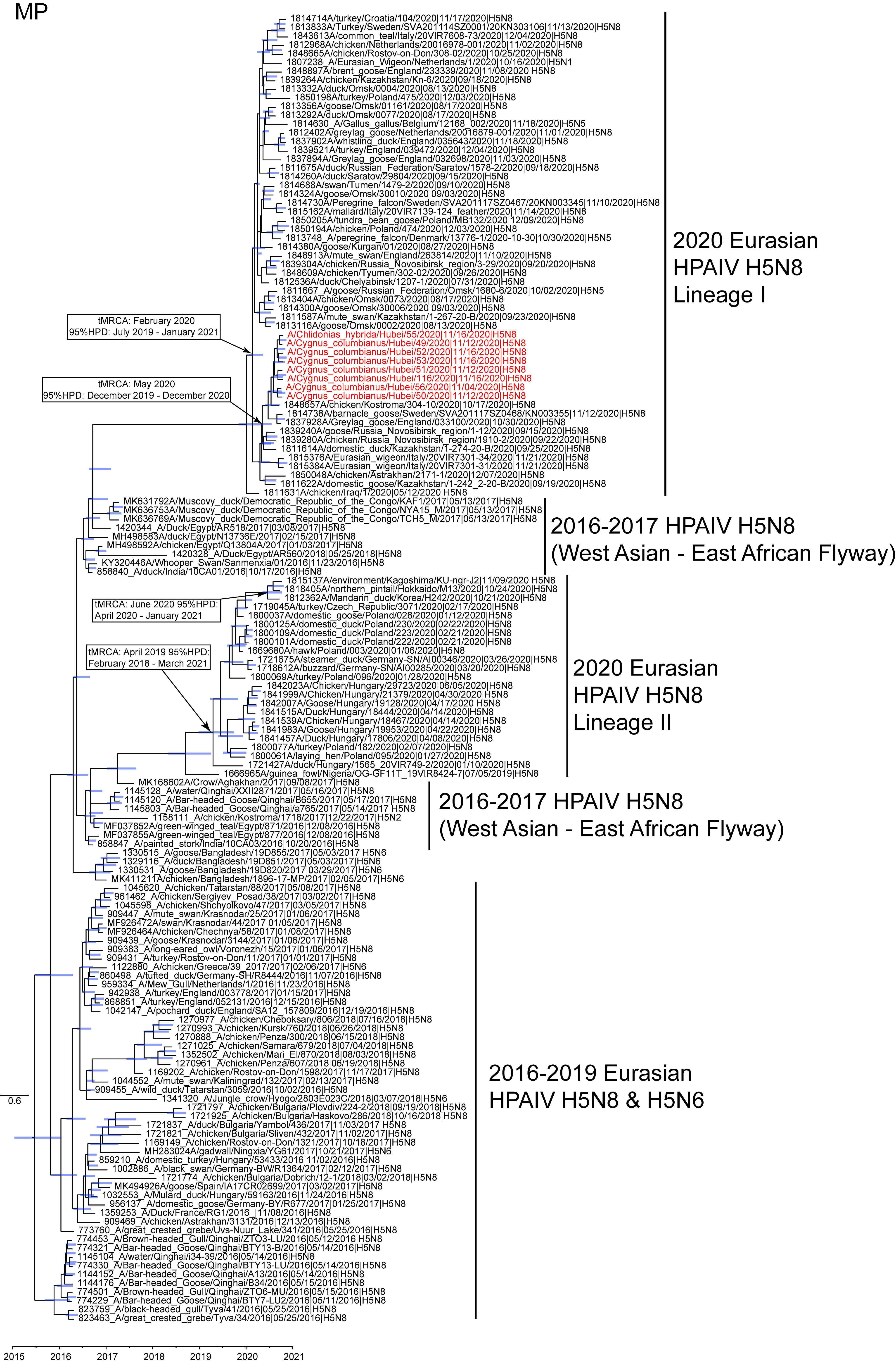

Supplement: Appendix_Figure_2G.tif [file TEMI_A_1956372_SM8251.tif]

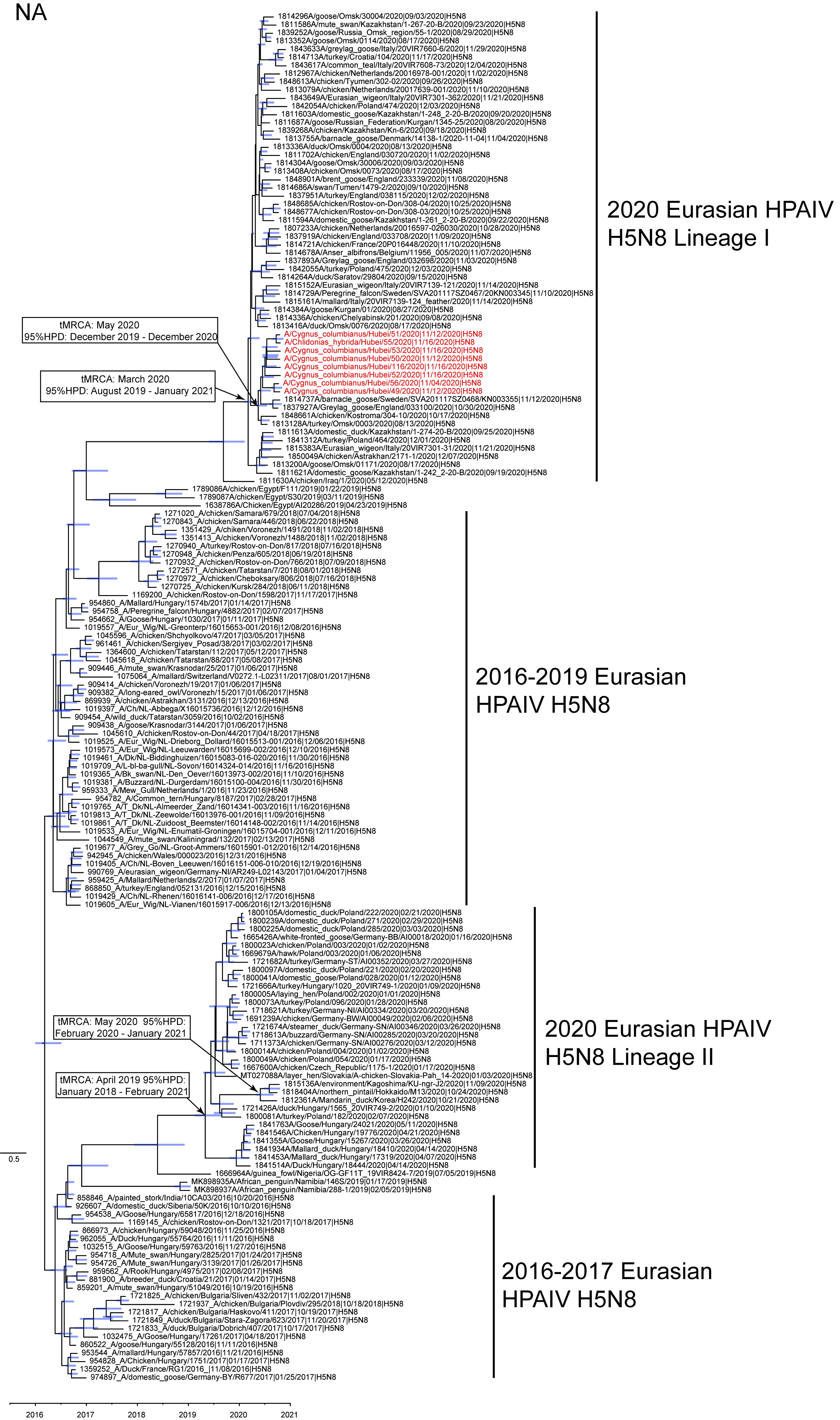

Supplement: Appendix_Figure_2F.tif [file TEMI_A_1956372_SM8250.tif]

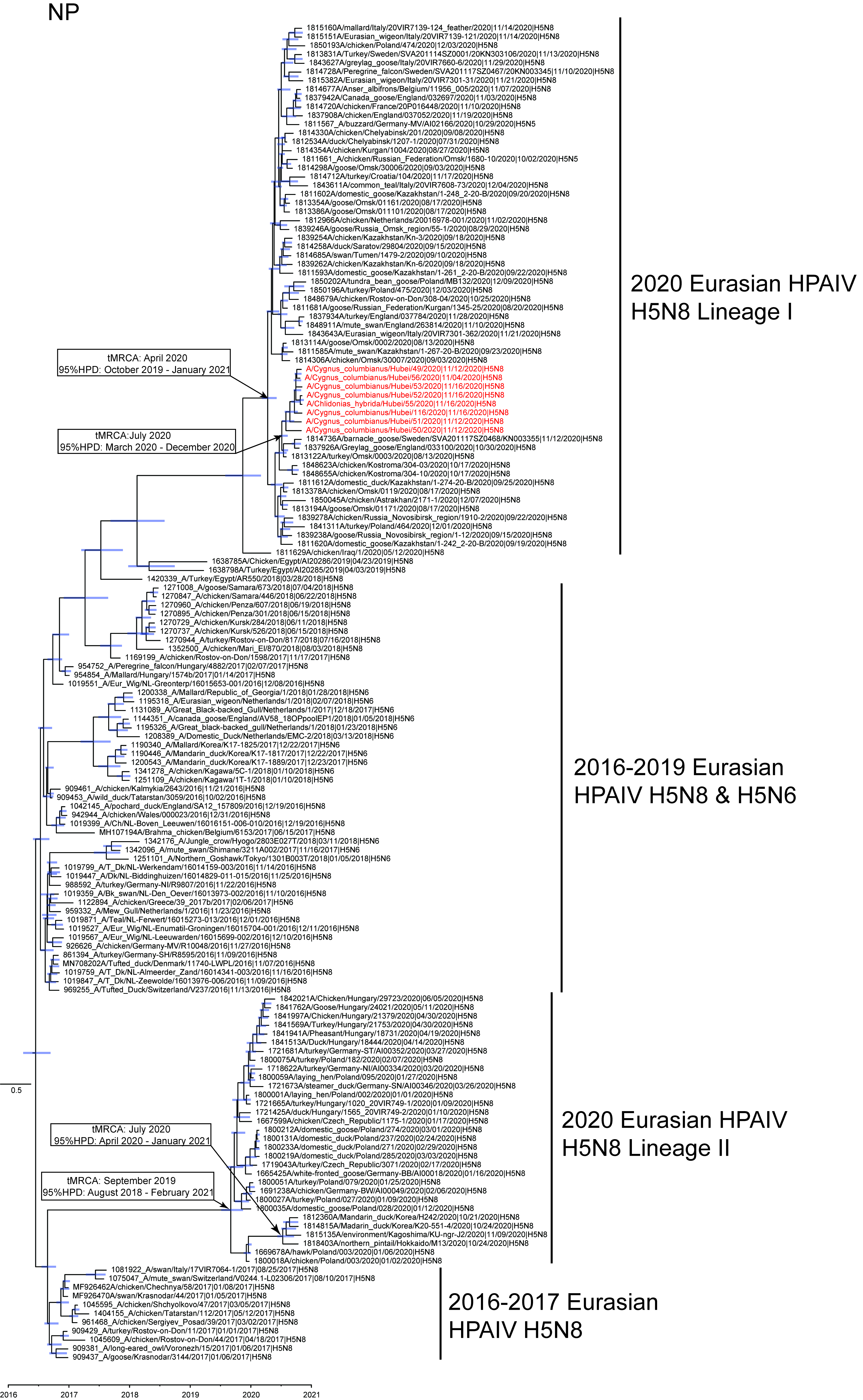

Supplement: Appendix_Figure_2E.tif [file TEMI_A_1956372_SM8249.tif]

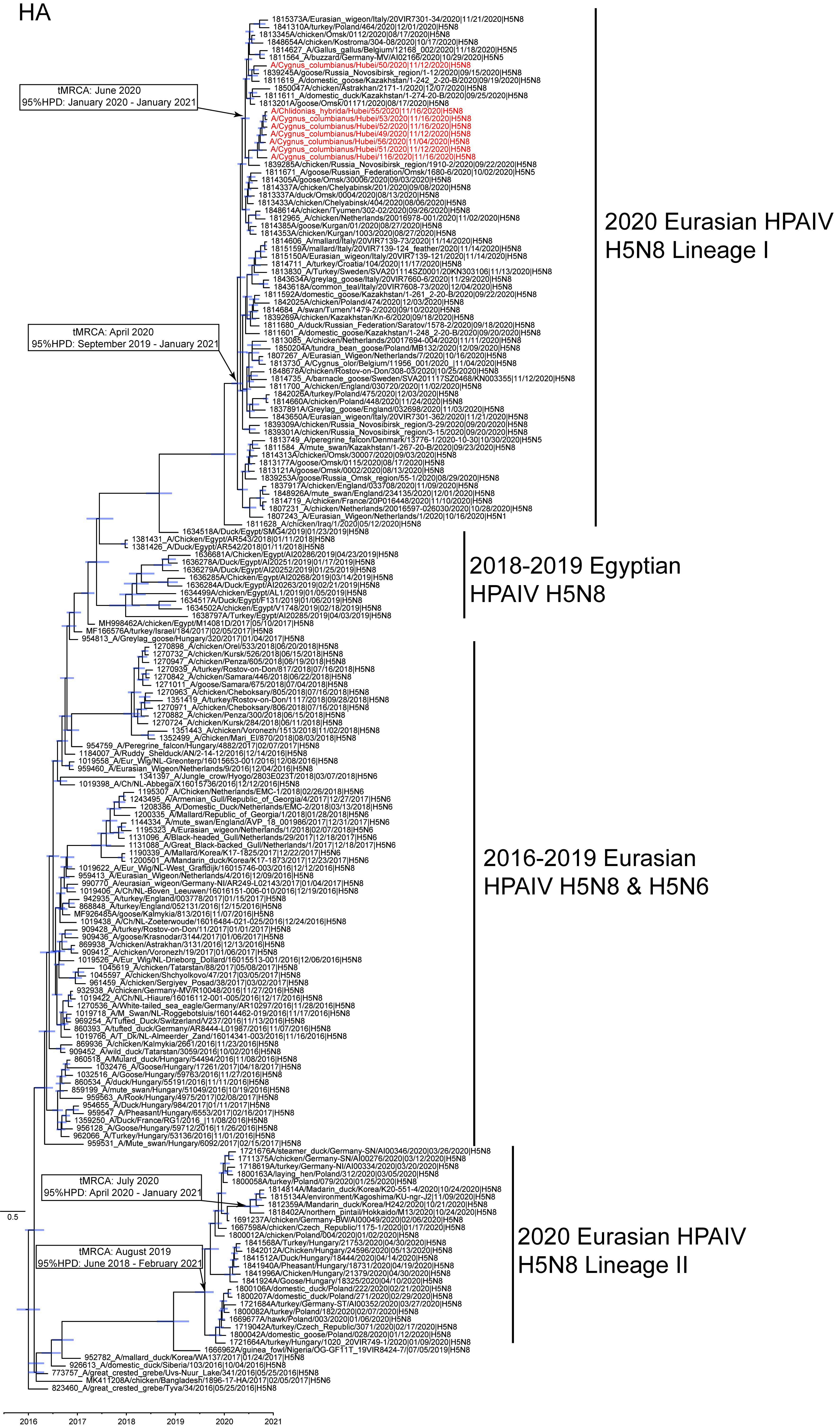

Supplement: Appendix_Figure_2D.tif [file TEMI_A_1956372_SM8248.tif]

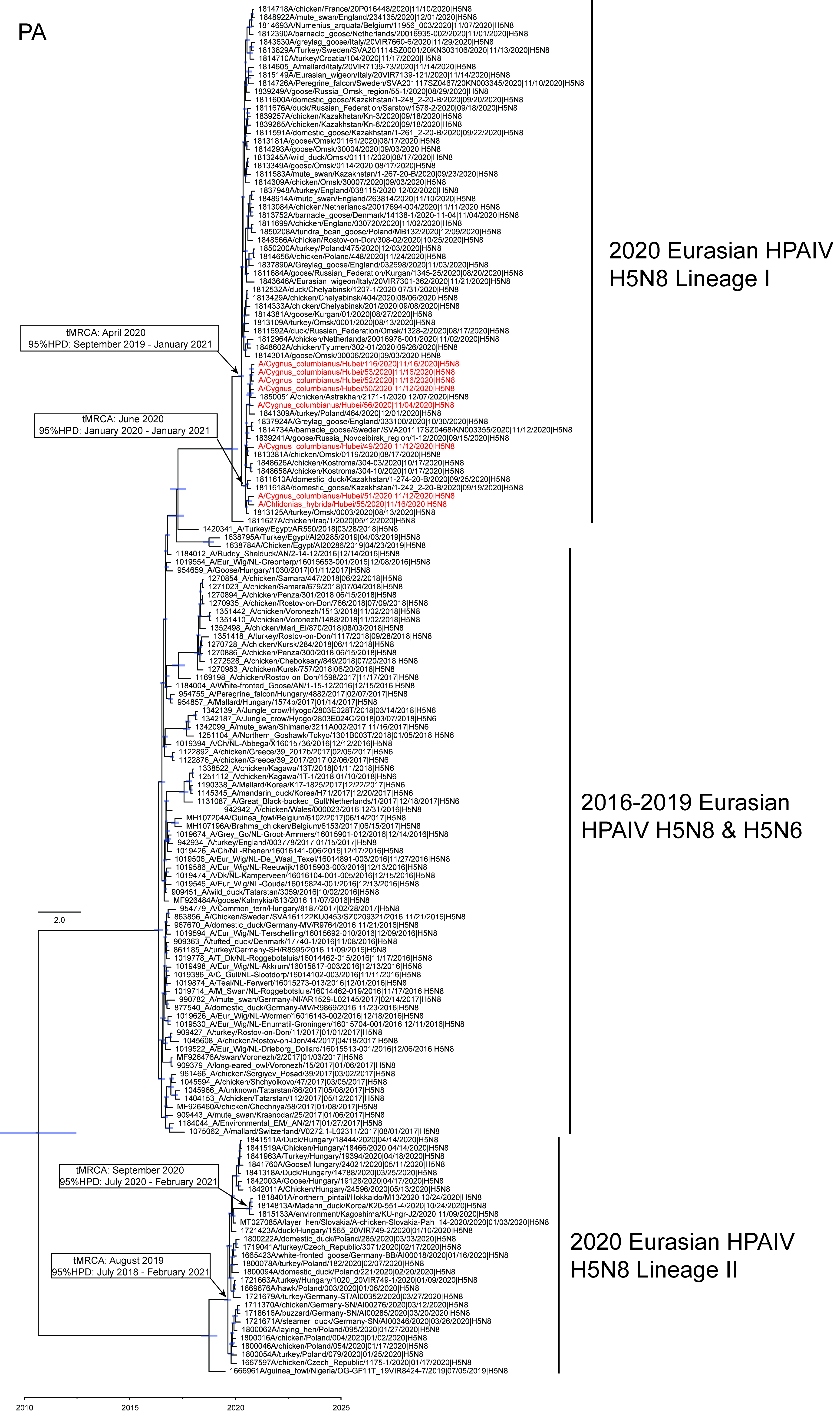

Supplement: Appendix_Figure_2C.tif [file TEMI_A_1956372_SM8247.tif]

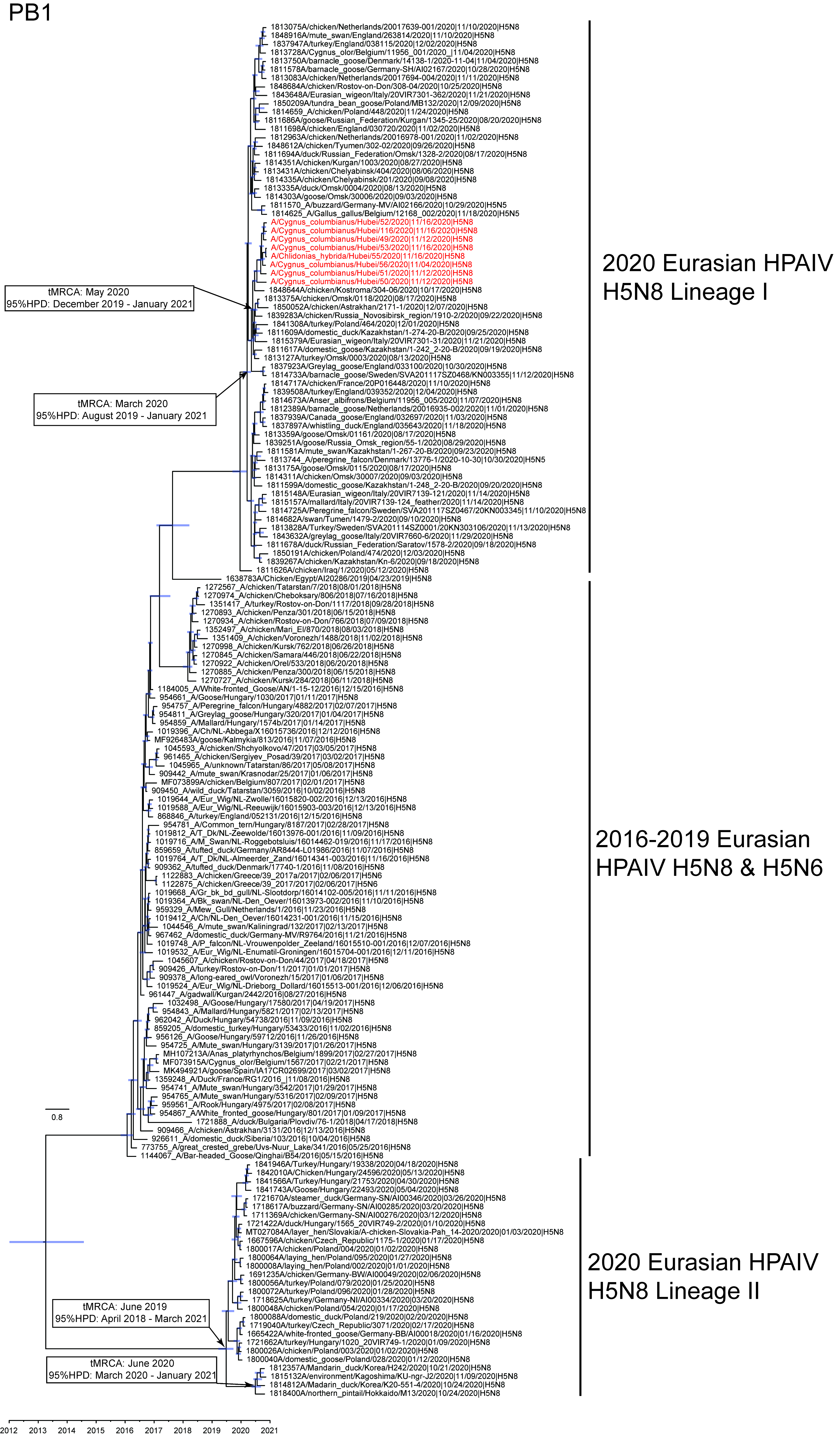

Supplement: Appendix_Figure_2B.tif [file TEMI_A_1956372_SM8246.tif]

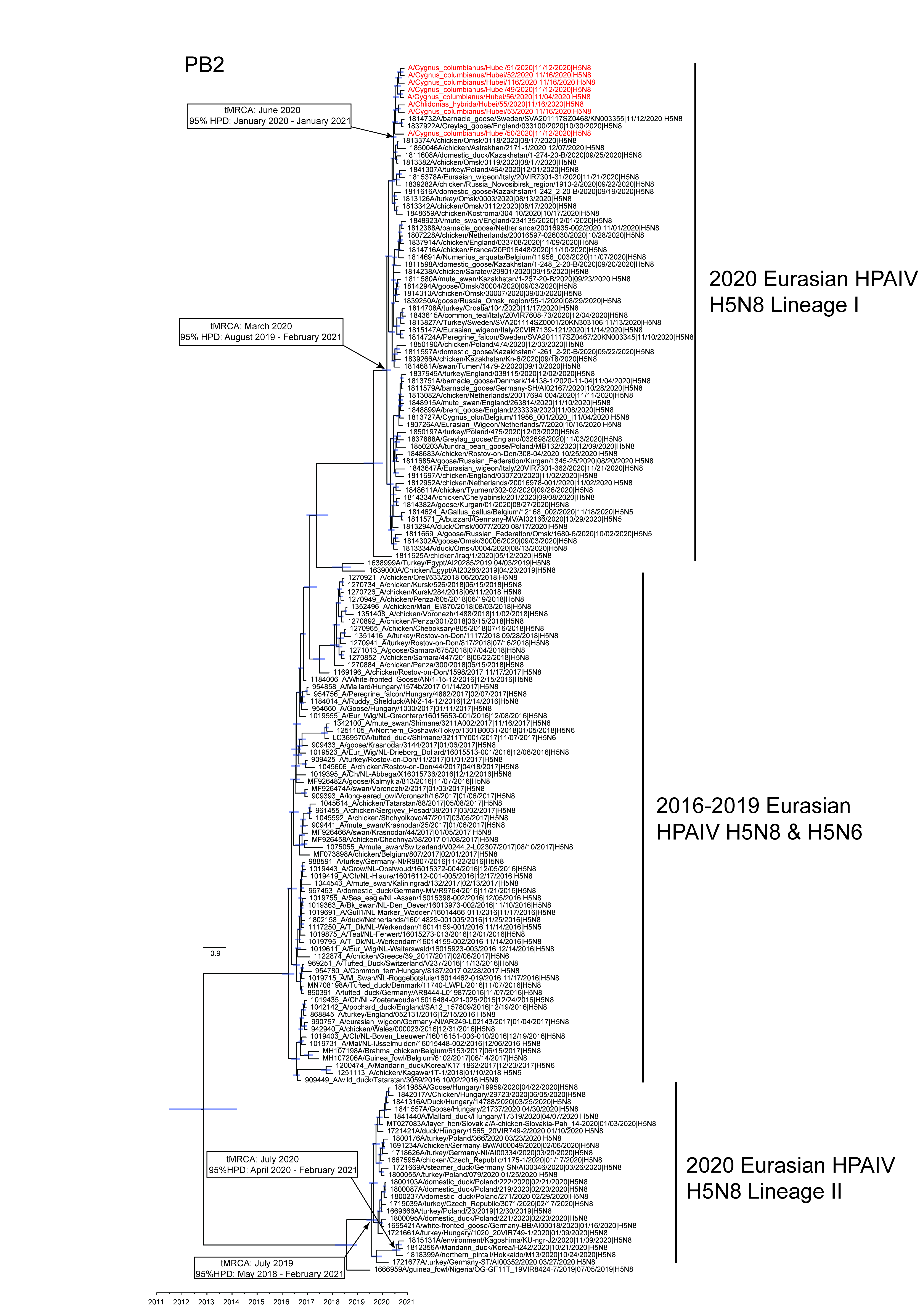

Supplement: Appendix_Figure_2A.tif [file TEMI_A_1956372_SM8245.tif]

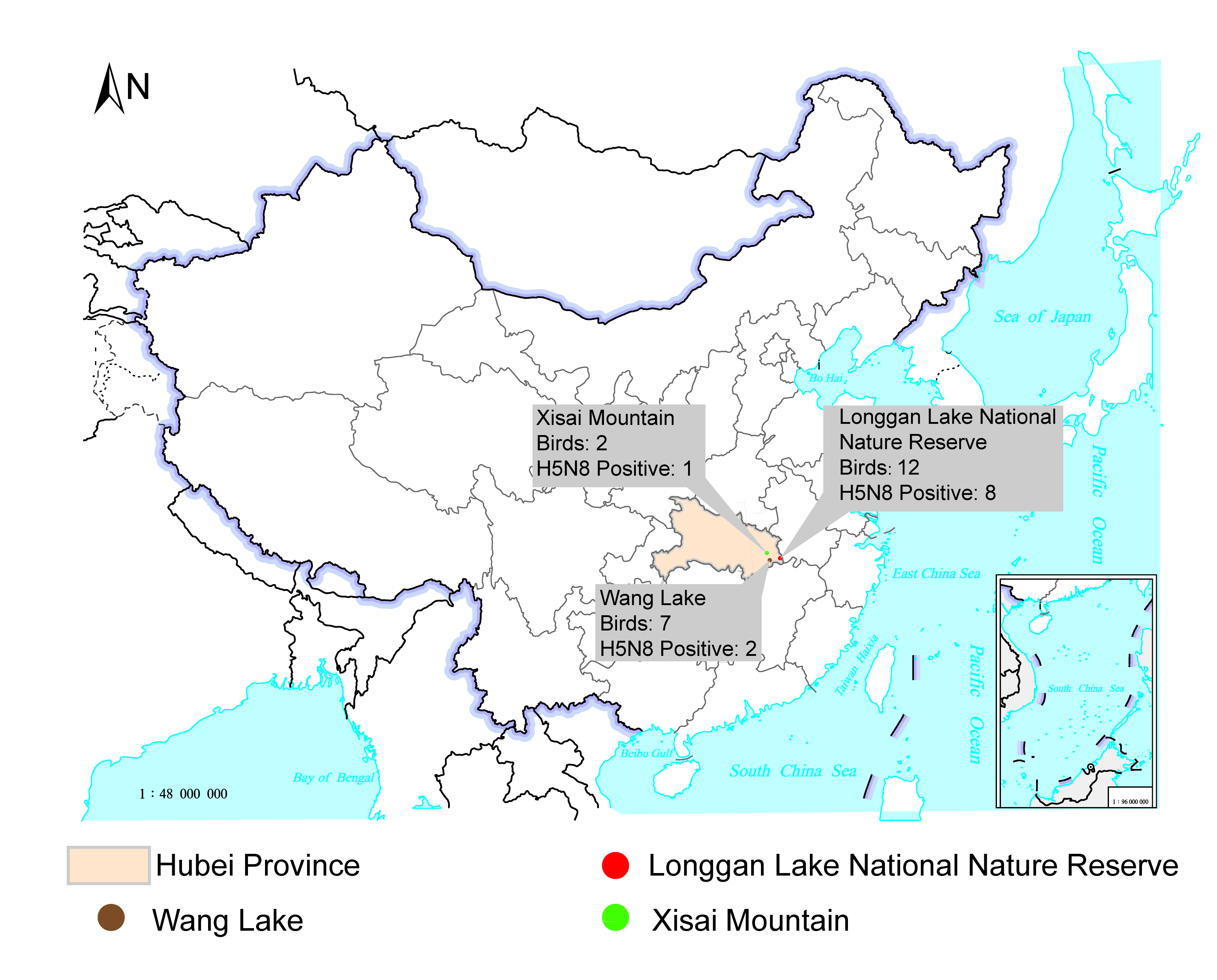

Supplement: Appendix_Figure_1.tif [file TEMI_A_1956372_SM8244.tif]
