# Supplementary material for: Emerging highly pathogenic avian influenza (H5N8) virus in migratory birds in Central China, 2020
Source: Emerg Microbes Infect. 2021 Jul 30;10(1):1503–6. doi: 10.1080/22221751.2021.1956372 (PMC8330791; doi:10.1080/22221751.2021.1956372)
Supplement: Appendix_Table_3.docx [file TEMI_A_1956372_SM8243.docx]

**Appendix Table 3**. Key molecular markers of the eight H5N8 viruses in the study

| Protein | Amino acid^†^/motif | Phenotypic consequences | References |
| --- | --- | --- | --- |
| HA^*^ | Cleavage site (PLREKRRKR/G) | Polybasic cleavage motif sequence required for high pathogenicity of H5N1 avian influenza viruses | [15,16] |
|  | T160A | Increased virus binding to α-2,6-linked sialic acid receptor ; increased transmission in guinea pigs | [17] |
| PB2 | L89V, G309D, T339K, R477G, I495V, K627E, A676T | Increased virulence  and replication in mammals. | [18] |
| PB1 | S622G | Increased polymerase activity and virulence in mice | [19] |
| PA | S515T | Increased polymerase activity in mammalian cells | [20] |
| M1 | N30D | Increased virulence in mice | [21] |
|  | T215A | Increased virulence in mice | [21] |
|  | I43M | Increased virulence in mice, chickens and ducks | [22] |
| NS1 | P42S | Increased virulence in mice | [23] |
|  | V149A | Increased virulence and decreased interferon response in chickens | [24] |
|  | C138F | Increased replication in mammalian cells, decreased interferon response | [25] |
|  | L103F, I106M | Increased virulence in mice | [26,27] |

^*^The numbering of HA is relative to A/New York/392/2004(H3N2).

^†^The numbering of each amino acid /motif (except HA) is relative to A/goose/Guangdong/1/1996(H5N1); All the eight H5N8 possess these amino acid /motifs.
