# Supplementary material for: Emerging highly pathogenic avian influenza (H5N8) virus in migratory birds in Central China, 2020
Source: Emerg Microbes Infect. 2021 Jul 30;10(1):1503–6. doi: 10.1080/22221751.2021.1956372 (PMC8330791; doi:10.1080/22221751.2021.1956372)
Supplement: Appendix_Table_2.docx [file TEMI_A_1956372_SM8242.docx]

**Appendix Table 2.** The highest nucleotide similarity of the eight H5N8 viruses in this study, with the sequences from the global initiative on sharing all influenza data (GISAID) and National Center of Biotechnology Information (NCBI)

| Gene | Viruses with the highest nucleotide identity | Accession number | Homology (%) |
| --- | --- | --- | --- |
| PB2 | A/barnacle goose/Sweden/SVA201117SZ0468/KN003355/2020(H5N8) | EPI1814732 | 99.15-99.83 |
| PB1 | A/barnacle goose/Sweden/SVA201117SZ0468/KN003355/2020(H5N8) | EPI1814733 | 99.40-99.66 |
| PA | A/Peregrine falcon/Sweden/SVA201117SZ0467/20KN003345/2020(H5N8) | EPI1814726 | 99.42-99.78 |
| HA | A/turkey/England/038115/2020(H5N8) | EPI1837949 | 99.44-99.72 |
| NP | A/Eurasian_wigeon/Italy/20VIR7139-121/2020(H5N8) | EPI1815151 | 99.36-99.49 |
| NA | A/turkey/England/038115/2020(H5N8) | EPI1837951 | 99.66-99.86 |
| MP | A/barnacle goose/Sweden/SVA201117SZ0468/KN003355/2020(H5N8) | EPI1814738 | 99.81-99.90 |
| NS | A/chicken/England/037052/2020(H5N8) | EPI1837911 | 99.21-99.78 |

Note: Each segment of the eight strains in the study shared the highest nucleotide identity to the virus as shown in the table.
