# Supplementary material for: Emerging highly pathogenic avian influenza (H5N8) virus in migratory birds in Central China, 2020
Source: Emerg Microbes Infect. 2021 Jul 30;10(1):1503–6. doi: 10.1080/22221751.2021.1956372 (PMC8330791; doi:10.1080/22221751.2021.1956372)
Supplement: Appendix_Table_1.docx [file TEMI_A_1956372_SM8241.docx]

**Appendix Table 1**. H5N8 viruses were detected in migratory birds from Nov 4, 2020 to Dec 10, 2020, in Central China

| Location | Collection date | Birds information | | | | | | | | | | | |
| --- | --- | --- | --- | --- | --- | --- | --- | --- | --- | --- | --- | --- | --- |
|  |  | Tundra swans  (*Cygnus columbianus*) | | | Bean geese  (*Chlidonias hybrida*) | | | Whiskered tern  (*Chlidonias hybrida*) | | | In total | | |
|  |  | Total | Dead | Rescued^†^ | Total | Dead | Rescued | Total | Dead | Rescued | Total | Dead | Rescued |
| Longgan Lake National Nature Reserve | 2020/11/4-2020/11/16 | 10 (7)^*^ | 7 (6) | 3 (1) | 1 | 0 | 1 | 1 (1) | 1 (1) | 0 | 12 (8) | 8 (7) | 4 (1) |
| Wang Lake | 2020/11/12-2020/11/30 | 5 (1) | 4 (1) | 1 | 2 (1) | 2 (1) | 0 | 0 | 0 | 0 | 7 (2) | 6 (2) | 1 |
| Xisai Mountain | 2020/11/20-2020/12/10 | 2 (1) | 2 (1) | 0 | 0 | 0 | 0 | 0 | 0 | 0 | 2 (1) | 2 (1) | 0 |
| Total | 2020/11/4 - 2020/12/10 | 17 (9) | 13 (8) | 4 (1) | 3 (1) | 2 (1) | 1 | 1 (1) | 1 (1) | 0 | 21 (11) | 16 (10) | 5 (1) |

^*^Figures in parentheses indicate the number of wild birds with H5N8 positive.

^†^Of the five rescued birds, one tundra swan with H5N8 positive was dead, and the other four rescued birds were later released to the wild (H5N8 negative) after they recovered.
