# Supplementary material for: Emerging highly pathogenic avian influenza (H5N8) virus in migratory birds in Central China, 2020
Source: Emerg Microbes Infect. 2021 Jul 30;10(1):1503–6. doi: 10.1080/22221751.2021.1956372 (PMC8330791; doi:10.1080/22221751.2021.1956372)
Supplement: Revised_Appendix_Clean.docx [file TEMI_A_1956372_SM8240.docx]

**Emerging highly pathogenic avian influenza (H5N8) virus in migratory birds in Central China, 2020**

**Appendix**

**Sample collection**

From November 4 to December 10 in 2020, dead or seriously sick migratory birds had been found in succession by the monitoring center of wildlife diseases and resources of Hubei province in several nature reserves in Hubei province, Central China. In total, 21 migratory birds were collected, including 17 tundra swans (*Cygnus columbianus*), three bean geese (*Anser fabalis*) and one whiskered tern (*Chlidonias hybrida*). In detail, 12 birds (eight dead and four rescued) were collected in Longgan Lake National Nature Reserve (longitude: 116.026703ºE, latitude: 29.986308ºN) between November 4 and November 16, 2020. Seven birds (six dead and one rescued) were collected in Wang Lake (longitude: 115.298682ºE, latitude: 29.903734ºN) between November 12 and November 30, 2020. Two birds (two dead) were collected in Xisai Mountain (longitude: 115.298682ºE, latitude: 30.210695ºN) on November 4 and December 10, 2020, respectively (Appendix Table 1). Some organs of 16 dead birds were dissected and oropharyngeal and cloacal swabs were collected from five rescued birds by the monitoring center of wildlife diseases and resources of Hubei province, then these samples were sent to our laboratory.

**Avian influenza virus identification**

The lung, liver, heart, spleen and kidney from dead migrated birds were ground, and 1 mL Trizol (Aidlab Biotechnologies Co., Ltd, BeiJing, China) was added into approximate 100mg of tissue, respectively. Then the tissues were completely lysed and homogenized. The lysates were transferred into 1.5ml EP tubes, and 200 ul trichloromethane was added. The tubes were vortexed until fully emulsified and allowed to stand for 3 min at room temperature. The tubes were then centrifuged at 12,000 rpm for 15min at 4℃. Then, 200ul supernatant was transferred into a new nuclease-free 1.5ml EP tube, and 200ul isopropanol was added. The solutions were gently mixed and RNA was precipitated at -30°C for 1h. After that, the tubes were centrifuged at 12,000 rpm for 15min at 4℃ and the supernatant was discarded. The RNA was washed with 1ml of 70% ethanol. The tubes were then centrifuged at 12,000 rpm for 15min at 4℃ and the supernatant was discarded. Then, 30ul nuclease-free water was added to dissolve RNA. One-step real-time reverse transcription-polymerase chain reaction (rRT-PCR) assay (Vazyme Biotech Co., Ltd. NanJing, China) was used to detect influenza A virus according to the [World Health Organization](http://www.baidu.com/link?url=kijvxk8G4gvj9gcIoWI3AjQqP5j-W1XLTjCVCCdf_Eu&wd=&eqid=f26f8305000107e500000003600a929b) guidelines[1]. The subtype was identified using H5 and N8 specific primers as described previously [2].

**Next generation sequencing (NGS)**

The cDNA sequences of eight confirmed H5N8 positive viruses were amplified using specific primers (primer F: 5’-ACGCGTGATCAGCAAAAGCAGG-3’, primer R: 5’-ACGCGTGATCAGTAGAAACAAGG-3’) and the products were sequenced using NGS [2]. Briefly, sequencing libraries were generated using an MGIEasy DNA Adapters-96 (Plate) kit (MGI Tech Co., Ltd, ShenZhen, China) following the manufacturer’s recommendations, and index codes were added. The library quality was assessed on a Qubit 3.0 Fluorometer (Life Technologies, Grand Island, NY, USA) and an Agilent 4200 system (Agilent, Santa Clara, CA, USA). The library was sequenced on the MGISEQ-2000 (PE150) platform.

**Sequencing data assembly**

The raw NGS reads were processed by filtering out low-quality reads (10 bases with quality <10), adaptor-contaminated reads (with > 15 bp matched to the adapter sequence), poly-Ns (with eight Ns), duplication, and host contaminated reads (SOAP2 version 2.21; less than five mismatches) [3]. The filtered reads were subjected to de novo assembly using Megahit (v1.2.9) [4], and the assembled contigs were blasted in the influenza database (downloaded from NCBI on December 2, 2020) to search for the best-matched reference sequences. The filtered reads were subsequently mapped to the selected reference sequences. Burrows-Wheeler Aligner (BWA version 0.7.17) [5] and SAMtools (version 1.9) [6] were then used to perform reference-based assembly.

**Phylogenetic analyses**

Sequences of all influenza A viruses from the GISAID and GeneBank databases were downloaded to create a local influenza A virus database (updated to December 10, 2020). Blastn was performed locally with default parameters against the local influenza A virus database, using each segment of the eight H5N8 viruses in the study as a query. Subsequently, the first 250 gene sequences in the output were collected. To investigate the relationship between the H5N8 detected in South Korea and Japan from October to November in 2020 and the strains we detected in the study. Together with the first 250 blastout sequences, H5N8 sequences in NCBI and GISAID collected from January 1, 2019 to December 10, 2020 were downloaded and integrated into one dataset with the gene sequences in this study. As a result, 8 datasets were obtained corresponding to eight segments of influenza A virus. Sequences in each segment dataset were aligned using MAFFT (v7.407) [7]. After this, the gene coding sequence was trimmed from the alignment to create a new dataset for each of the eight segments with duplicate sequences removed to construct the maximum likelihood and maximum clade credibility (MCC) phylogenetic trees. The maximum likelihood tree of the hemagglutinin (HA) gene was generated using RAxML-HPC2 (v.8.2.12) [8] on XSEDE in CIPRES [9] with the GTRGAMMA substitution model and 1,000 bootstrap iterations.

To investigate the source of these viruses in our study, we constructed the MCC phylogenetic trees. In detail, first, aligned sequences of each segment were analyzed by Tempest (v1.5.3)[10] to make sure there was a temporal signal. GTR nucleotide substitution model with a four-category gamma distribution model (GTR+F+G4 nucleotide substitution model) was selected using ModerFinder [11] in Akaike Information Criterion (AIC). Under GTR+F +G4 nucleotide substitution model and chain length of 200,000,000, different molecular clocks (strict and uncorrelated relaxed clock) and tree priors (constant size, exponential growth, logistic growth, expansion growth, GMRF bayesian skyirde, and bayesian skyline) were tested using path sampling/stepping-stone sampling[12] (for which we employed 20 path steps with length chains of 5,000,000) to estimate the (log) marginal likelihood to select the most suitable model. Eventually, by comparing Bayes Factors[13] of different clocks and tree priors, GTR+F+G4 nucleotide substitution model and strict clock with bayesian skyline tree prior were chosen for the analysis using beast v 1.10.4[14]. The reliability of the results was confirmed using Tracer (v1.7.1) (all Ess values were above 200). We performed two independent runs for all eight segments datasets, and similar convergence was observed to make sure the reliability of the results. The MCC trees were summarized using TreeAnnotator (v1.10.4), with 10% burn-in cutoffs. All maximum-likelihood phylogenetic trees and MCC trees were visualized using FigTree (v1.4.3).

**Histopathology**

Lung, liver, heart, spleen, and kidney were collected from dead migratory birds (tested positive for H5N8 by RT-PCR). These tissues were fixed in 10% neutral-buffered formalin and embedded in paraffin. Hematoxylin and eosin staining was performed on sections of these tissues to evaluate the histopathology.

Immunohistochemical staining was performed on sections of lung to detect and evaluate the expression of the influenza A virus antigen. Briefly, the lung was fixed in 10% neutral-buffered formalin, embedded in paraffin and cut into sections (4um). Then, sections were deparaffinized with xylene and alcohol. Subsequently, sections were heated in citrate buffer (pH 6.0) for antigen retrieval and incubated with 3% H_2_O_2_ to block the endogenous peroxidase. Sections were incubated with 3% bovine serum albumin (BSA) in PBS for 30 minutes to block nonspecific binding sites. After that, the sections were incubated with a 1:500 dilution of mouse antibody against nucleoprotein of influenza A virus overnight at 4℃. And the sections were subsequently incubated with horseradish peroxidase (HRP)–conjugated rabbit anti-mouse immunoglobulin G (IgG) (a secondary antibody) for 1h at 37℃. Then, sections were stained with 3, 3 -diaminobenzidine (DAB) and hematoxylin. Images were acquired using a Panoramic scanner (3D-Histech, Hungary).

**References**

1. World Health Organization. WHO information for molecular diagnosis of influenza virus - update. [Internet]. 2015 May [cited 2021 Jan 30]. Available from: https://www.who.int/influenza/gisrs_laboratory/molecular_diagnosis_influenza_virus_humans_update_201403rev201505.pdf?ua=1

2. Bi Y, Chen Q, Wang Q, et al. Genesis, Evolution and Prevalence of H5N6 Avian Influenza Viruses in China. Cell Host Microbe. 2016 Dec 14;20(6):810-821.

3. Li R, Yu C, Li Y, et al. SOAP2: an improved ultrafast tool for short read alignment. Bioinformatics. 2009 Aug 1;25(15):1966-7.

4. Li D, Luo R, Liu CM, et al. MEGAHIT v1.0: A fast and scalable metagenome assembler driven by advanced methodologies and community practices. Methods. 2016 Jun 1;102:3-11.

5. Li H, Durbin R. Fast and accurate short read alignment with Burrows-Wheeler transform. Bioinformatics. 2009 Jul 15;25(14):1754-60.

6. Li H, Handsaker B, Wysoker A, et al. The Sequence Alignment/Map format and SAMtools. Bioinformatics. 2009 Aug 15;25(16):2078-9.

7. Katoh K, Standley DM. MAFFT multiple sequence alignment software version 7: improvements in performance and usability. Mol Biol Evol. 2013 Apr;30(4):772-80.

8. Stamatakis A. RAxML version 8: a tool for phylogenetic analysis and post-analysis of large phylogenies. Bioinformatics. 2014 May 1;30(9):1312-3.

9. Miller MA, Pfeiffer, W., Schwartz, T. (2010) "Creating the CIPRES Science Gateway for inference of large phylogenetic trees" in Proceedings of the Gateway Computing Environments Workshop (GCE), 14 Nov. 2010, New Orleans, LA pp 1 - 8.

10. Rambaut A, Lam TT, Max Carvalho L, et al. Exploring the temporal structure of heterochronous sequences using TempEst (formerly Path-O-Gen). Virus Evol. 2016 Jan;2(1):vew007.

11. Kalyaanamoorthy S, Minh BQ, Wong TKF, et al. ModelFinder: fast model selection for accurate phylogenetic estimates. Nat Methods. 2017 Jun;14(6):587-589.

12. Baele G, Lemey P, Bedford T, et al. Improving the accuracy of demographic and molecular clock model comparison while accommodating phylogenetic uncertainty. Mol Biol Evol. 2012 Sep;29(9):2157-67.

13. Kass RE, Raftery AE. Bayes Factors. Journal of the American Statistical Association. 1995 1995/06/01;90(430):773-795.

14. Suchard MA, Lemey P, Baele G, et al. Bayesian phylogenetic and phylodynamic data integration using BEAST 1.10. Virus Evol. 2018 Jan;4(1):vey016.

15. Schrauwen EJA, Herfst S, Leijten LM, et al. The multibasic cleavage site in H5N1 virus is critical for systemic spread along the olfactory and hematogenous routes in ferrets. Journal of virology. 2012;86(7):3975-3984.

16. Suguitan AL, Jr., Matsuoka Y, Lau Y-F, et al. The multibasic cleavage site of the hemagglutinin of highly pathogenic A/Vietnam/1203/2004 (H5N1) avian influenza virus acts as a virulence factor in a host-specific manner in mammals. Journal of virology. 2012;86(5):2706-2714.

17. Gao Y, Zhang Y, Shinya K, et al. Identification of amino acids in HA and PB2 critical for the transmission of H5N1 avian influenza viruses in a mammalian host. PLoS pathogens. 2009 Dec;5(12):e1000709.

18. Li J, Ishaq M, Prudence M, et al. Single mutation at the amino acid position 627 of PB2 that leads to increased virulence of an H5N1 avian influenza virus during adaptation in mice can be compensated by multiple mutations at other sites of PB2. Virus Res. 2009 Sep;144(1-2):123-9.

19. Feng X, Wang Z, Shi J, et al. Glycine at Position 622 in PB1 Contributes to the Virulence of H5N1 Avian Influenza Virus in Mice. Journal of virology. 2016 Feb 15;90(4):1872-9.

20. Hulse-Post DJ, Franks J, Boyd K, et al. Molecular changes in the polymerase genes (PA and PB1) associated with high pathogenicity of H5N1 influenza virus in mallard ducks. Journal of virology. 2007 Aug;81(16):8515-24.

21. Fan S, Deng G, Song J, et al. Two amino acid residues in the matrix protein M1 contribute to the virulence difference of H5N1 avian influenza viruses in mice. Virology. 2009 Feb 5;384(1):28-32.

22. Nao N, Kajihara M, Manzoor R, et al. A Single Amino Acid in the M1 Protein Responsible for the Different Pathogenic Potentials of H5N1 Highly Pathogenic Avian Influenza Virus Strains. PLoS One. 2015;10(9):e0137989.

23. Jiao P, Tian G, Li Y, et al. A single-amino-acid substitution in the NS1 protein changes the pathogenicity of H5N1 avian influenza viruses in mice. Journal of virology. 2008 Feb;82(3):1146-54.

24. Li Z, Jiang Y, Jiao P, et al. The NS1 gene contributes to the virulence of H5N1 avian influenza viruses. Journal of virology. 2006 Nov;80(22):11115-23.

25. Li J, Zhang K, Chen Q, et al. Three amino acid substitutions in the NS1 protein change the virus replication of H5N1 influenza virus in human cells. Virology. 2018 Jun;519:64-73.

26. Kuo RL, Krug RM. Influenza a virus polymerase is an integral component of the CPSF30-NS1A protein complex in infected cells. Journal of virology. 2009 Feb;83(4):1611-6.

27. Spesock A, Malur M, Hossain MJ, et al. The virulence of 1997 H5N1 influenza viruses in the mouse model is increased by correcting a defect in their NS1 proteins. Journal of virology. 2011 Jul;85(14):7048-58.

**Figure legends**

Appendix Figure 1. Geographical distribution of the sampling locations of 21 migratory birds in Hubei province, Central China. Wang Lake, Xisai Mountain, and Longgan Lake National Nature Reserve are three nature reserves in Hubei province.

Appendix Figure 2. The maximum clade credibility (MCC) trees of eight H5N8 viruses in the study. (A) polymerase basic (PB2). (B) polymerase basic (PB1). (C) polymerase (PA). (D) hemagglutinin (HA). (E) nucleoprotein (NP). (F) neuraminidase (NA). (G) matrix (MP). (H) nonstructural protein (NS). The H5N8 viruses reported in the study are highlighted in red. The MCC trees were constructed using the BEAST v1.10.4, with the GTR nucleotide substitution with a four-category gamma distribution model and a strict clock with bayesian skyline tree prior. Node bars indicate 95% highest posterior density (95% HPD) of the node height. The time to the most recent common ancestor (tMRCA) with 95% HPD is labeled in the important nodes. HPAIV: highly pathogenic avian influenza virus

Appendix Figure 3. Pathological changes and quantification of viral load in the lung, liver, heart, spleen, and kidney in dead migratory birds infected with H5N8. (A)Pathological changes in lung. Inflammatory cell infiltration (yellow arrowheads), erythrocyte (yellow arrows). (B) Liver, inflammatory cell (read arrowheads) near the central vein (CV) and edema in the space of Disse (red arrows) were observed. (C) Myocarditis in the heart. Inflammatory cell infiltration (green arrowheads) and necrosis of the myofibers (green arrows) (D) Spleen, lymphocyte depletion was obvious, and necrosis of lymphocyte were observed (cyan arrowheads), amyloidosis (cyan arrows), LF (lymphoid follicle). (E) Pathological changes in kidney, necrosis of renal tubular epithelial cells (thick white arrows) was observed. Interstitial hemorrhage (thin white arrows) with inflammatory cell infiltration (white arrowhead) were obvious. (F) Viral load of highly pathogenic avian influenza virus (HPAIV) H5N8 in the lung, liver, heart, spleen, and kidney. The lung of 53, the liver of 116, and spleen of 55 were not detected.

52: A/Cygnus columbianus/Hubei/52/2020(H5N8);

53: A/Cygnus columbianus/Hubei/53/2020(H5N8);

55: A/Chlidonias hybrida/Hubei/55/2020(H5N8);

116: A/Cygnus columbianus/Hubei/116/2020(H5N8)

(G) Section of lung was stained by immunohistochemistry with a mouse antibody against the nucleoprotein of influenza A virus. (H) Higher magnification image of section of lung in (G).

Pictures of H&E stained tissue sections of lung, liver, heart, spleen and kidney were from tundra swans. There is no significant difference in histopathology of lung, liver, heart, spleen and kidney between tundra swan and whiskered tern. Note: Pathological changes in these organs shown in this figure are partly masked by postmortem autolytic changes, because these wild birds lived in nature, before they have been found dead, they had been dead for a long time.
